# Investigating Menzerath’s law in crows and humans during cued vocal ‘counting’

**DOI:** 10.1007/s10071-026-02054-4

**Published:** 2026-03-14

**Authors:** Diana A. Liao, Akseli Ilmanen, Katharina F. Brecht, Andreas Nieder

**Affiliations:** Animal Physiology, Institute of Neurobiology, University of Tübingen, Auf der Morgenstelle 28, 72076 Tübingen, Germany

**Keywords:** Vocal control, Linguistic laws, Comparative cognition, Numerosity, Corvids

## Abstract

To uncover universal principles of vocal behavior, studies have examined whether linguistic laws apply across species. Menzerath’s law, for instance, posits that larger constructs have shorter constituent parts, reflecting an efficiency principle that reduces articulatory and energetic demands. Another proposed universal is final lengthening, where end-of-sequence vocalizations are extended to potentially signal sequence boundaries. While studies have examined these temporal patterns in ecological communicative contexts, it remains unclear whether they also emerge in more constrained, externally instructed vocal responses. This study examines temporal patterns in vocal sequences during a numerically cued production task in crows and humans. While both species performed the same task, they differ in their numerical representations: crows rely on an approximate number system, whereas humans possess a symbolic understanding of number. Crows showed a negative correlation between sequence length and vocalization duration—consistent with Menzerath’s law—and a positive correlation between sequence position and duration, consistent with final lengthening. In contrast, humans exhibited the opposite: vocalization duration increased with sequence size and decreased with position. These findings suggest that Menzerath’s law is not a universal principle but shaped by species-specific cognition, motor constraints, and task demands. This highlights the importance of considering context and cognitive capacities when interpreting temporal patterns in vocal behavior across species.

**Supplementary Information:**

The online version contains supplementary material available at 10.1007/s10071-026-02054-4.

## Introduction

Identifying general phenomena shared between human language and non-human animal communication systems can help uncover the evolutionary mechanisms that shape fundamental biological organization across species. One such phenomenon is Menzerath’s law, which has been colloquially summed as ‘the longer the whole, the shorter its parts’, predicting a negative relationship between the size of a sequence and the size of its constituent elements (Menzerath 1954; Altmann and Grotjahn 1980). First identified in human speech across multiple linguistic levels, this pattern is thought to reflect a trade-off between communicative effectiveness and energetic constraints—by shortening elements in longer sequences, signalers reduce articulatory effort while maintaining information content (Semple et al. 2022, Torre et al. 2019).

Intriguingly, vocal patterns adhering to Menzerath’s law have also been observed in non-human animals, including the diverse communication systems of primates, whales, bats, and birds (Gustison et al. 2016; Fedurek et al. 2017; Huang et al. 2020; Valente et al. 2021; Youngblood 2025; Zhang et al. 2024). Amongst these, avian species studied include penguins, finches, and multiple other songbird species (Favaro et al. 2020; James et al. 2021; Youngblood 2024; Wascher and Youngblood 2025). These findings suggest that this temporal pattern may reflect a domain-general principle of efficiency—balancing communicative demands with production constraints across species.

However, whether Menzerath’s law represents a truly universal principle underlying communication across species remains an open question. Evidence from other animal species suggest its presence may be contingent on context, species-specific vocal constraints, or the unit of measurement (Clink and Lau 2020; Clink et al. 2020; Deng et al. 2024; Watson et al. 2020, 2024; James et al. 2021). Importantly, most studies have examined communicative contexts in the wild, where the sequence structure is presumably shaped by the signaler’s own communicative goals and thus internally generated. A promising approach to uncovering the constraints that shape Menzerath’s law is to investigate vocal sequences elicited under experimentally-controlled conditions where sequence size—the number of vocalizations—is determined by external instruction rather than by internal communicative intent. If Menzerath’s law primarily reflects biomechanical or energetic constraints, then we would expect it to hold even in such non-communicative contexts, across both humans and non-human animals trained to produce specific vocal sequence sizes. However, if Menzerath’s law is also shaped by communicative pressures, cognitive strategies, or species-specific vocal control, then deviations from the expected pattern may emerge in tasks where the goal is to match a numerical cue rather than convey meaning. Comparing species with different numerical systems and vocal control in a shared, constrained task offers a unique opportunity to test whether Menzerath’s law reflects universal constraints or is context- and species-dependent.

We test whether temporal patterns predicted by Menzerath’s law appear in externally-cued vocal sequences produced by crows (Liao et al. 2024) and humans. In the numerically-cued vocal production task, participants of both species were instructed – either verbally (humans) or via training with the cues (crows) to produce a specific number of vocalizations in response to neutral cues. These two lineages, who diverged over 320 million years ago, exhibit striking similarities in volitional vocal control and flexibility alongside other cognitive capabilities such as categorization, rule-switching, sequence generalization, numerical processing, self-control, tool use, and more (Brecht et al. 2019, Brecht et al. 2023, Liao et al. 2025, Liao et al. 2022, Wascher and Reynolds 2025, Wagener and Nieder 2023, Miller et al 2019, Veit and Nieder 2013, Taylor et al. 2010, Moll et al. 2025, Apostel et al. 2023, Güntürkün and Bugnyar 2016, Emery and Clayton 2004). By comparing species that differ in their numerical cognition – crows relying on approximate number representations whereas humans employ symbolic counting (Nieder 2018, 2021; Dehaene et al. 2022) – we also assess how cognitive representational systems for number shape temporal organization in vocal behavior.

The approximate number system is an evolutionarily ancient system for estimating the number of items in a set (Nieder 2021; Messina et al. 2020; Scarf et al. 2011; Chittka and Geiger 1995). The ANS allows estimation of all set sizes but becomes systematically less precise with increasing numerical value; this numerical size effect is one signature of the ANS. For young children who haven’t learned symbolic counting (Starkey and Cooper 1980; Feigenson et al. 2004) and adult populations that are unfamiliar with symbolic counting in their culture (Gordon 2004; Pica et al. 2004; Frank et al. 2008), their behavioral performance on numerical tasks suggests that the ANS is an elementary, noisy, nonverbal system for assessing set size. Building on the ANS with mathematical education, the representation of number becomes more exact while allowing for more elaborate numerical skills (Halberda et al. 2008; Dehaene et al. 2022). Thus far, humans are the only known species to have a fully symbolic understanding of number.

We also compare the uncovered patterns in the externally-cued vocal sequences of trained crows to crow vocal sequences from the wild collected by citizen scientists. Investigating whether Menzerath’s law manifests in such controlled settings, we aim to disentangle the contributions of cognitive, energetic, and articulatory constraints underpinning the production of vocal sequences across species.

## Methods

### Recordings of laboratory crows

Analysis of the crow recordings, in which they performed the numerically-cued vocal production task, was conducted using a pre-existing dataset (Liao et al. 2024). Participants were three male carrion crows (Corvus corone, age: 3 years) from the Institute of Neurobiology’s facility. Crows were kept on a controlled feeding protocol during experiments and earned food as a reward during training. Additional food was supplemented if necessary and water was provided ad libitum. All procedures were conducted in accordance with German and European law and approved by the national authority, the Regierungspräsidium Tübingen.

Experiments were conducted in a darkened chamber enclosed by sound-attenuating foam. For additional details on the experimental apparatus and training process, see Liao et al. (2024). To perform the numerically-cued vocal production task, crows self-initiated each trial by positioning their head before the touchscreen monitor. A vocalization cue appeared which instructed the production of a specific number of vocalizations. In this analysis, we included only visual cue trials (colored Arabic numerals), which were shared with the human experiments. The crows had to produce the cued number of vocalizations and then peck a confirmation stimulus (“enter key”) to indicate that they were done vocalizing. After this peck, the produced number of vocalizations was compared with the cued number. If they matched, the trial was correct and the crow was presented with a food reward. If they did not match, the trial was an error and the crow had a brief timeout. Presentation of stimuli and collection of behavioral responses were managed by the CORTEX system (National Institute for Mental Health, Bethesda, Maryland). Audio signals were recorded using a Sennheiser MKE 600 microphone placed above the touchscreen monitor with a 40,000 Hz sampling rate for vocalization detection and offline analysis of vocal features.

A custom MATLAB routine automatically detected the onset and offset of any acoustic signal that differed from the background noise using the envelope of the sound amplitude waveform. Manual verification of these putative vocalizations were used to confirm the timing data for further analysis. Initial experimental sessions were checked by two experimenters but after high agreement, the rest of the sessions were verified by a single experimenter. We include all vocalizations from all trials in the following analyses – both resulting in correct and error responses. A total of 11,997 vocalizations and 4620 sequences were used in this analysis. Bird 1 produced 2214 vocalizations and 928 sequences. Bird 2 produced 4705 vocalizations and 1799 sequences. Bird 3 produced 5078 vocalizations and 1893 sequences.

### Wild recordings of crows

To examine whether similar temporal patterns are present in carrion crow vocal sequences produced in the wild, we analyzed vocal recordings available on the citizen science databases xeno-canto.org and eBird.org. Recordings were first manually inspected through the interactive spectrogram viewer Chirpity (Kirkland 2025) and time periods in which a crow was vocalizing were exported as separate files. These exported files included metadata about the date and time of the recording, but not the location of recording or the ID of the database user. Since xeno-canto and eBird users occasionally upload multiple recordings on the same day which sometimes originate from the same date and location, potentially capturing the same crow multiple times, recordings from the same day and database were set to have a shared recording-ID. Assuming the same crows were very rarely recorded across multiple days, the number of recording-IDs acts as an approximate lower bound for the number of crows sampled.

From these longer manually-segmented periods, Whisperseg (Gu et al. 2024) was used to segment individual calls. The Whisperseg encoder model receives convolved spectrograms as input, and is thus sensitive to noisy spectrograms. Therefore, files were pre-screened and excluded if they had too high spectral flatness (Wiener entropy, > 0.05) which indicates broadband noise, and/or too high temporal entropy (> 0.85) which indicates a uniform power distribution across time rather than sharp onset/offset boundaries in the spectrogram. After Whisperseg segmentation, we manually inspected all segmentations next to their spectrograms and excluded sequences with inaccurate onset/offset or where multiple individuals were vocalizing simultaneously. Next, the durations of individual vocalizations were calculated and saved alongside information on the number of vocalizations in the sequence, the position of the vocalizations in the sequence, and the recording-ID. A list of the recording files is available in the Supplementary materials. From an initial 957 recording-IDs, 434 remained after exclusion criteria were applied. A total of 3143 vocalizations and 1115 sequences from these recording-IDs were used in this analysis.

### Recordings of humans

Twenty-six participants (aged: 21–39; 18 Women, 8 Men) were recruited and tested at the Institute of Biology at the University of Tübingen, Germany. The experiment was performed with approval of the Ethical Committee of the Faculty of Science, University of Tübingen and was in accordance with the ethical standards as laid down in the 1964 Declaration of Helsinki. A total of 18,174 vocalizations and 4054 sequences were used in this analysis. For individuals, the average number of vocalizations was 699 with a standard deviation of 72.24; the average number of sequences was 155.92 with a standard deviation of 16.3.

Testing was performed in the same sound-attenuated experimental chamber and with the same program as the trained crows. We initially planned to use the same numerical range (i.e. 1–4) as the crows but pilot testing on DAL and KFB showed that performance in this range was at ceiling (i.e. 100% accuracy). To potentially have some error trials to analyze, we extended the range of numerical cue stimuli to 1 – 8. Another difference is that humans were not rewarded after every correct trial as they did not need continuous motivation to complete trials but received a small food reward at the end of the testing session. We do not expect this change in reward structure to affect performance on this task. Instead of a sequence of training steps over the course of months, participants were instructed verbally that the purpose of the experiment was to examine interactions between numerosity and vocal production so they were told to produce a certain number of vocalizations to visual cues. However, they were not informed about the exact relationships or hypotheses to be tested. After the numerical cue appears, they should produce the cued number of vocalizations and then manually-tap the touchscreen to end the trial. As we did not have strong hypothesis on the exact sound the humans should produce, for comparative purposes, they were instructed to make ‘caw’ or ‘ka’ sounds. Trials were self-paced similar to the crows and each session took around 15 minutes in total.

### Statistical analysis

The model used to test Menzerath’s law (modified from Altmann and Grotjahn 1980; Torre et al. 2021) is a linear model with the log-transformed element duration as the dependent variable, the log-transformed sequence size (SeqSize) and the position in the sequence (Position) as the fixed effects. Included in the model are random intercepts for sequence identity ($${u}_{j})$$ to account for repeated measures within sequences, individual subject (BirdID, $${v}_{k}$$) to account for individual differences, and ordinal position treated as a category (PositionCat, $${w}_{l}$$) to account for random associations of vocalizations with particular positions. Models were fit using the fitlme function in MATLAB (Ver. 2020b, Mathworks, USA).

$$\begin{aligned}\mathrm{log}\left({CallDuration}_{ijk}\right)&={\beta }_{0}+{\beta }_{1}\mathrm{log}\left({SeqLength}_{ijk}\right)\\&+ {\beta }_{2}{Position}_{ijk}+{u}_{j}+{v}_{k}+{w}_{l}+{\epsilon }_{ijk}\end{aligned}$$

The same model is run for the dataset of vocal sequences from wild crows and humans. For the wild crows and humans, the random effect BirdID was replaced with RecordingID and HumanID respectively. We report the beta weights for the effect of Sequence Size and Sequence Position alongside the corresponding standard error, the t-statistic, and the p-value.

To test for differences between the vocal sequences of trained and wild crows, we fit the model above with an additional effect of Context. We report the Context x SeqSize and Context x Position interactions.

To test for differences between the vocal sequences of trained crows and humans, we fit the model above with an additional effect of Species. We report the Species x SeqSize and Species x Position interactions.

### Removal of single vocalizations

Linguistic studies on Menzerath’s law have often excluded single-element sequences (i.e., a length of one) as a single element is equivalent to the entire sequence (Torre et al. 2019, 2021; Semple et al. 2022). This consideration is highlighted by a study of Menzerath’s law in the ‘close call’ sequences produced by mountain gorillas where the negative relationship found between number of vocalizations and sequence length was driven entirely by the difference between single- and multi-unit sequences (Watson et al. 2020). For completeness, we report models including and excluding single vocalizations.

### Production constraint model

Patterns conforming to Menzerath’s law (i.e. negative relationship between number and duration of vocalizations) can sometimes be detected in random sequences (James et al. 2021; Ferrer-i-Cancho et al. 2014; Tanaka-Ishii 2021). An alternate approach is to simulate pseudorandomized sequences of syllables that match the durations of real sequences but can vary in the number of syllables. The slope of these pseudorandomized syllable durations and sequence sizes can differ from the real slope calculated from the dataset and could be interpreted as indicating additional mechanisms over simple motor constraints. To calculate a chance distribution, we follow the production constraint model of James et al. (2021). For each sequence in the data, a pseudorandom sequence was produced. Vocalizations were randomly sampled from each crows’ distribution until the duration of the random sequence exceeded the duration of the real sequence. If the total duration of random sequence exceeded the real sequence by more than 50% of the duration of the last vocalization, the last vocalization was removed. Else, the sequence was accepted. With this process, the generated random sequences had approximately the same distribution of durations as the real data. The vocal durations, sequence size, and position in sequence for these generated sequences were used to fit the same lme model to get a distribution of slopes.

It has been suggested that this is too conservative of a null model as analyses of human language datasets do not show steeper slopes than the chance distribution (Youngblood 2025).

We also calculate a null model without production constraints by shuffling the vocalization durations within crows. We then fit the LMM model to these randomized sequences to get a distribution of slopes.

### Correlation between durations of first and last vocalizations and sequence size

To test for a relationship between sequence size and the durations of the first and last vocalizations, we used the MATLAB function corrcoef to calculate the Pearson correlation coefficient. We report the r and p values.

### Comparison of last vocalizations to non-last vocalizations

Following the procedure in Tierney et al. (2011) for analyzing the phrase-final durations in human music and in birdsong, we computed the mean duration of all vocalizations in each sequence. Dividing by this mean duration, the relative durations of individual vocalizations were then computed. For each sequence, we then calculated the mean relative duration of final and non-final calls and compared these using a paired t-test with sequences as the unit of analysis.

### Accuracy of humans in numerically-cued vocal production task

To test how if accuracy varies across numbers in humans, we fit a linear model with the Accuracy (calculated as percent correct over all trials) as the dependent variable and the cued number (CueNum) as the fixed effect. We also included random intercepts for each participant and random slopes for the effect of cue number by participant.

We report the beta weights for the effect of CueNum alongside the corresponding standard error, the t-statistic, and the p-value.

## Results

### Temporal patterns in crow cued vocal sequences

**Fig. 1:**
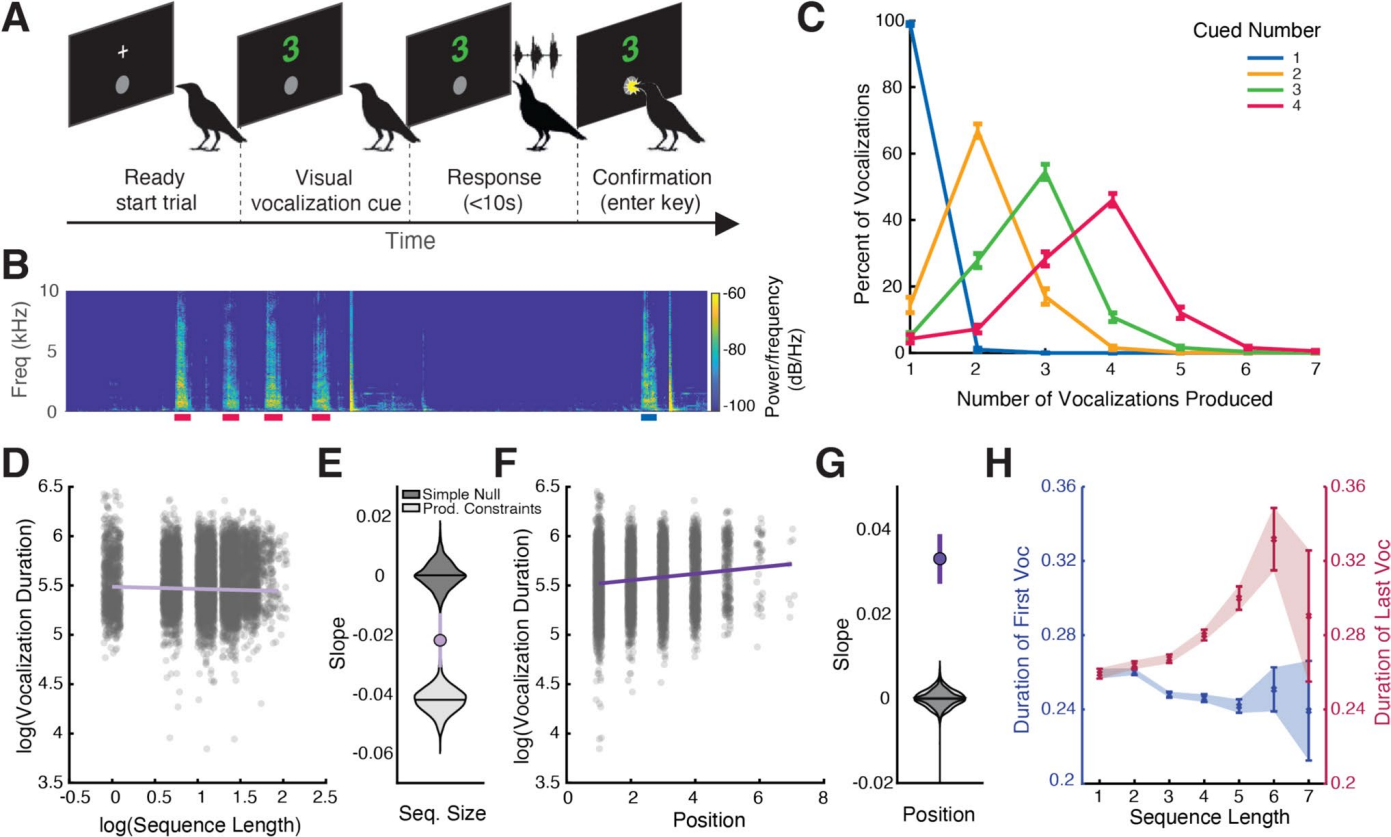
Temporal patterns in numerically-cued vocal sequences of trained crows. **A)** Structure of numerically-cued vocal production task. **B)** Example spectrogram of 2 trials from a crow. Color bars indicate the durations of the identified vocalizations with colors indicating the cued number. **C)** Behavioral performance of all three crows. Color bars indicate the cued number and error bars indicate the standard error of the mean (SEM). **D)** Relationship between sequence size and vocal durations. A linear mixed effect model was fit to the crows’ vocal durations with predictors of sequence size and position in the sequence. The slope of the model corresponding to the relationship with sequence size is overlaid over individual vocalizations shown as jittered dots. **E)** Results from Monte Carlo simulations. Violin plots depict the distribution of slopes expected by chance (lighter grey: simple null model, darker grey: production constraint model) with the circle indicating the observed slope. **F)** Relationship between position in sequence and vocal durations. From the same model as above in **A**, the slope corresponding to the relationship with position in sequence is overlaid over individual vocalizations shown as jittered dots. **G)** Results from Monte Carlo simulations for slope of position and sequence size. **H)** Examining the durations of the first vocalizations (in blue on left Y-axis) in a sequence for every sequence size, we find a negative relationship with first vocalization duration and increasing sequence size. In contrast, this relationship between the last vocalization durations (in red on right Y-axis) and sequence size is positive. Shaded error bars represent the SEM

Crows were trained to produce a specific number of vocalizations to neutral visual cues (Fig. 1A). The order of the presented numerical cues were randomized; an example spectrogram containing the vocal sequences of two trials is shown in Fig. 1B. Crows were able to successfully perform the task and the bell-shaped behavioral performance curves demonstrate signatures of the approximate number system (Fig. 1C**,** see Liao et al. 2024).

Taking all vocal sequences, we test for adherence to Menzerath’s law by examining the relationship between the duration of individual vocalizations and sequence size. We fit a linear mixed effects model and found that the duration of vocalizations was significantly and negatively related to the number of vocalizations in the sequence (β = -0.022 ± 0.005, t = -4.69, p < 0.0001, Fig. 1D). As negative relationships between the duration and number of vocalizations could arise by chance, we also test two null hypotheses using Monte Carlo simulations. First, a simple randomization by shuffling vocal durations within birds produced slopes centered around zero (Fig. 1E). Second, following James et al. 2021, we tested whether these patterns could arise from production constraints alone by constructing pseudorandom sequences that match the cumulative duration in time. Surprisingly, the slope distribution of these simulations were even more negative than our observed slope (Fig. 1E). As some previous studies have excluded single vocalizations (Torre et al. 2019; Watson et al. 2020), we fit the model without single vocalizations and still find a negative relationship (β = -0.042 ± 0.007, t = -5.87, p < 0.0001).

One process by which Menzerath’s law could arise is by shortening vocalizations towards sequence ends and having more short vocalizations with longer sequences. So, we examined the correlation between the duration of individual vocalizations and position in the sequence. We find a positive relationship (β = 0.034 ± 0.003, t = 11.00, p < 0.0001, Fig. 1F) where vocalizations get longer then further along the sequence the vocalization is made. The slope observed was more positive than expected by chance (Fig. 1G).

To investigate how the durations of individual vocalizations exhibit both a negative correlation with sequence size and a positive correlation with position in a sequence, we plot the durations of the first and last vocalization in a sequence by the sequence size (Fig. 1H). We find a negative correlation where the first vocalizations in a sequence get shorter the higher the number of total vocalizations (r = -0.09, p < 0.0001). Interestingly, there is a positive correlation with the duration of the last vocalizations in a sequence and the number of vocalizations (r = 0.12, p < 0.0001).

To test for final lengthening, we compute relative vocal durations by dividing each vocalization's duration by the mean duration in its sequence. The sequence-final vocalizations were significantly longer than non-final vocalizations (t(3,576) = 7.67, p < 0.0001).

### Comparison with crow vocal sequences in the wild

**Fig. 2:**
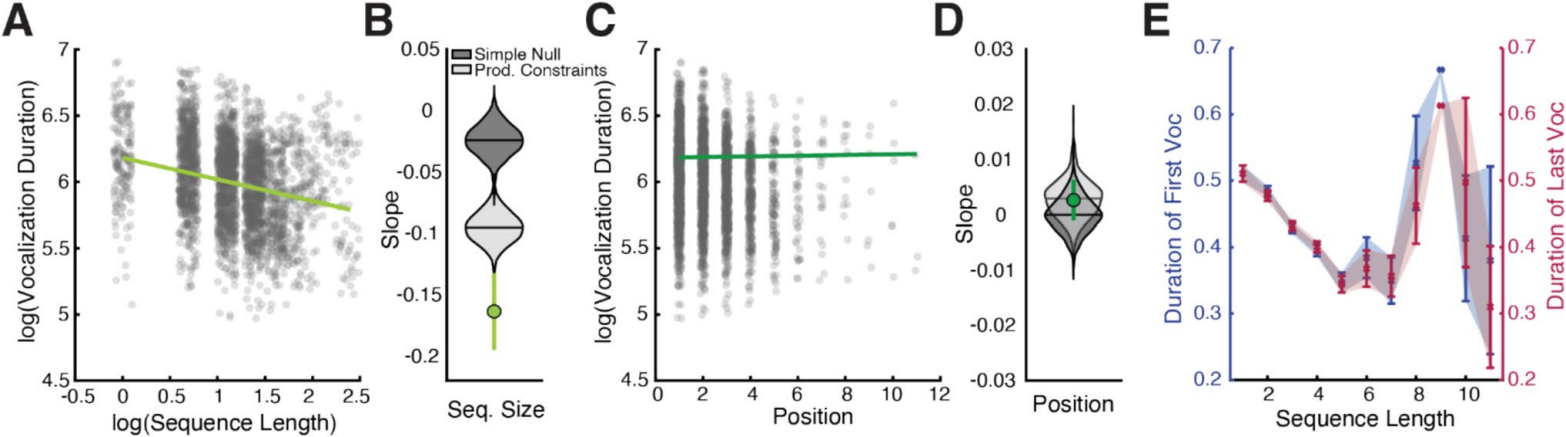
Temporal patterns in crow vocal sequences in the wild. **A)** In wild crow vocal sequences, there is a negative slope (in light green) between vocal duration and sequence size. Individual vocalizations are shown as jittered dots. **B)** This slope is more negative than expected from a chance distribution calculated with Monte Carlo simulations (lighter grey: simple null model, darker grey: production constraint model). **C)** The slope (in dark green) corresponding to the relationship between vocal duration and position in sequence is overlaid over individual vocalizations. **D)** The slope for position in sequence is not different than expected from chance. **E)** Examining the durations of the first vocalizations (in blue on left Y-axis) in a sequence for every sequence size, we find a negative relationship with first vocalization duration and increasing sequence size. This relationship is very similar for the last vocalization durations (in red on right Y-axis) and sequence size

The comparison of Menzerath’s law in crow vocal sequences from the wild can be used to dissociate how temporal patterns are shaped by externally-cued sequence lengths versus internally-generated sequence lengths. We fit the same model to recordings taken from online repositories xeno-canto.org and ebird.org. We find a strong negative relationship between the vocal durations and the number of vocalizations in the sequence (β = -0.164 ± 0.016, t = -10.27, p < 0.0001, Fig. 2A). This slope was more negative than expected from chance (Fig. 2B). Interestingly, we find no significant correlation between the vocal durations and position in the sequence (β = 0.003 ± 0.002, t = 1.40, p = 0.16, Fig. 2C**/D**). These relationships were similar when single vocalizations were removed (Sequence Size: β = -0.199 ± 0.024, t = -8.47, p < 0.0001, Position in Sequence: β = 0.003 ± 0.002, t = 1.44, p = 0.15).

Consistent with these patterns, both the first and the last vocalizations in the sequence are negatively correlated with sequence size (first vocalization: r = -0.27, p < 0.0001, last vocalization: r = -0.28, p < 0.0001). The negative correlation for the first vocalization indicates that crows might adjust the vocalization duration based on intended sequence length. The correlation for the last vocalization confirms that this pattern persists throughout the sequence (Fig. 2E). The relationships between vocal durations with sequence size and ordinal position differ between the trained and wild crow sequences (Context x log_SeqLength: β = 0.065 ± 0.006, t = 10.24, p < 0.0001; Context x Position: β = 0.016 ± 0.002, t = 10.25, p < 0.0001).

### Temporal patterns in human cued vocal sequences

**Fig. 3:**
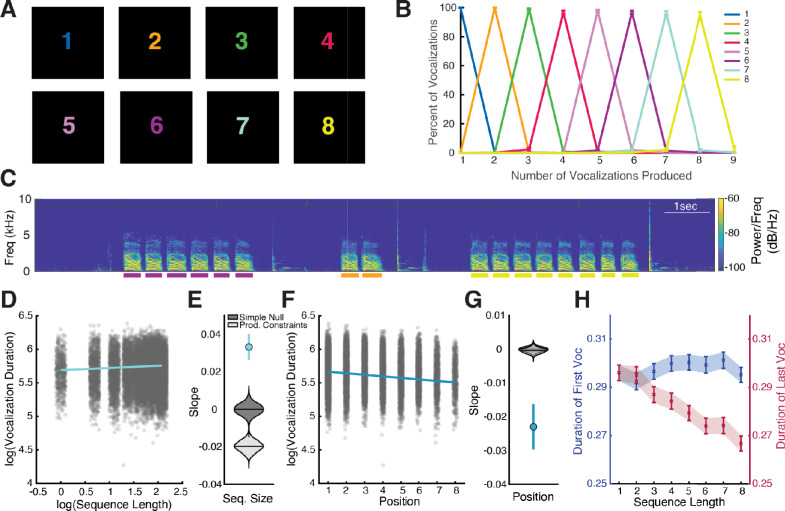
Temporal patterns in numerically cued vocal sequences of humans. **A)** Stimuli used for humans in the numerically-cued vocal production task. **B)** Behavioral performance of 26 humans. Color lines indicate the cued number and error bars indicate the standard error of the mean (SEM). **C)** Example spectrogram of 3 trials from a human. Color bars indicate the durations of the identified vocalizations with colors indicating the cued number. **D)** A linear mixed effect model was fit to the humans’ vocal durations with predictors of sequence size and sequence position. The slope of the model corresponding to the relationship with sequence size is overlaid over individual vocalizations shown as jittered dots. **E)** The slope for sequence size is more positive than expected from chance. **F)** Relationship between position in sequence and vocal durations. **G)** The slope for position in sequence is more negative than expected from chance. **H)** Examining the durations of the first vocalizations (in blue on left Y-axis) in a sequence for every sequence size, we find very similar vocal durations across sequence sizes. In contrast, we find a negative relationship between the last vocalization durations (in red on right Y-axis) and increasing sequence size

Humans performed the same numerically-cued vocal production task as the crows with an increased range of numbers from 1 to 8 (Fig. 3A). Humans very successfully performed the task, demonstrating above 95% accuracy on every number tested (Fig. 3B)**.** Even with such high performance, there was a decrease in accuracy with increasing number, declining from 99.8% for 1 vocalization to 94.9% for 8 vocalizations (main effect of ‘cue number’: β = -0.007 ± 0.002, t = -3.58, p = 0.0004). An example spectrogram containing the vocal sequences of three trials from an example human is shown in Fig. 3C.

In contrast to crows, humans performing the cued vocal production task showed an opposite pattern. Vocalization duration increased with sequence size (β = 0.033 ± 0.004, t = 9.32, p < 0.0001, Fig. 3D). The slope of the relationship between duration and size observed was more positive than expected by chance (Fig. 3E). Additionally, when examining the position in the sequence, vocal duration decreased with later positions (β = -0.023 ± 0.003, t = -6.73, p < 0.0001, Fig. 3F). This slope of the relationship between duration and position was more negative than expected by chance (Fig. 3G). These relationships were similar when single vocalizations were removed (Sequence Size: β = 0.059 ± 0.005, t = 12.29, p < 0.0001, Position in Sequence: β = -0.023 ± 0.004, t = -6.61, p < 0.0001).

Correlating the durations of the first vocalization in the sequence and sequence size (r = 0.02, p = 0.267), reveal that the durations of the first vocalization in a sequence do not vary significantly with sequence size (Fig. 3H). However, a negative correlation between the durations of the last vocalization in the sequence and sequence size (r = -0.13, p < 0.0001) reveal that vocalizations get shorter with (longer sequences (Fig. 3I). The task-based vocal sequences of these humans and our trained crows demonstrate significantly different relationships with sequence size and ordinal position (Species x log_SeqLength: β = 0.034 ± 0.003, t = 12.65, p < 0.0001; Species x Position: β = -0.020 ± 0.001, t = -21.36, p < 0.0001).

## Discussion

Our study reveals striking differences in how crows and humans organize vocal sequences when externally cued to produce specific numbers of vocalizations in a controlled task. In trained crows, we observed a negative relationship between sequence size and vocalization duration, consistent with Menzerath’s law. However, Monte Carol simulations (James et al. 2021)—where call durations where randomly shuffled while preserving sequence durations – revealed that this relationship was weaker than expected from production constraints alone. Intriguing, this negative relationship between sequence size and vocalization duration was present even more strongly in wild crow vocal sequences, exceeding the distributions calculated from simulations. This contrast suggests that compression effects are modulated by context – whether sequences are internally initiated in communicative settings or externally cued in cognitive tasks. In contrast, humans exhibited an opposite pattern in the externally-cued task; Vocalization durations increased with sequence size. This pattern suggests that external task demands interact with distinct cognitive strategies in humans; symbolic numerical understanding and explicit counting strategies may fundamentally reshape vocal temporal organization.

These results raise important questions about the universality of linguistic laws in vocal communication. While Menzerath’s law has been reported across diverse species and communicative contexts (Gustison et al. 2016; Fedurek et al. 2017; Huang et al. 2020; Valente et al. 2021; Youngblood 2025; Zhang et al. 2024; Favaro et al. 2020; James et al. 2021), our findings highlight that its expression is not uniform. Even within a single species, the strength of the pattern varies depending on whether sequences are internally-initiated in the wild or externally-cued in a cognitive task**.** As these temporal patterns are statistical tendencies rather than absolute laws, exceptions are not completely unexpected and can be used to uncover alternative factors and context-dependence (Semple et al. 2022). The attenuation of the compression effect in trained crows suggests the presence of external cues and task demands may engage different cognitive and motor systems than wild crows balancing communicative demands—such as signal optimization and energetic constraints.

### Context-dependence of final lengthening

One process by which Menzerath’s law could arise is by shortening vocalizations towards sequence ends and having more short vocalizations with longer sequences. This is thought to reflect energetic or breathing constraints (Gustison et al. 2016; MacLarnon and Hewitt 1999; Suthers et al. 1999). We find the opposite; the trained crows increased the duration of their vocalizations with ordinal position. Additionally, final vocalizations are significantly longer than non-final vocalizations. This pattern is consistent with ‘Final lengthening’ where vocalizations tend to slow or expand at sequence boundaries (Tierney et al. 2011, Oller 1973; Paschen et al. 2022; Wightman et al. 1992). In human language, final lengthening is a widespread feature of prosody, aiding in receiver segmentation of the speech stream, and facilitating vocal turn-taking). It been described for Germanic, Slavic, Romance, Uralic, Afro-Asiatic, Indo-European languages as well as numerous linguistic families from the Americas, Africa, and Asia. Interestingly, it has also been described for music and manual gestures (Todd 1985; Weismer and Ingrisano 1979; Fenlon et al. 2019). In animal communication systems, patterns consistent with final lengthening has also been observed in bird song (Tierney et al. 2011; James et al. 2021) and the vocal sequences of other species (Watson et al. 2024; Huang et al. 2020; Valente et al. 2021). This phenomenon is thought to reflect both motoric constraints (e.g. the slowing of articulatory gestures before stopping) and communicative functions that enhance signal clarity at boundaries. We speculate that the goal-directed constraint of producing a specific number of vocalizations may lead to boundary marking as a cognitive strategy as the trained crows organize the sequence of vocalizations as a whole.

### Cognitive mechanisms: sequence planning versus serial counting

The combination of relationships between vocal durations, sequence size, and position in sequence support that finding that when externally-cued, crows plan the vocal sequence as a whole before production. In previous analyses to examine planning, we found a strong positive correlation between reaction time and the number of vocalizations and that the acoustic features of the very first vocalization in the sequence can be used to predict the total number of subsequent vocalizations (Liao et al. 2024). This vocal planning allows for temporal compression characteristic of Menzerath’s law, though the effect is attenuated compared to wild contexts, possibly due to the cognitive load of tracking progression to the target number. The contrasting results in humans suggest that symbolic numerical understanding and task demands may override compression effects observed in naturalistic speech and language. When humans use symbolic counting to track sequence length (counting ‘one, two, three’ internally), each vocalization is processed as an individual unit rather than part of a planned whole. This serial, one-at-a-time strategy would differ fundamentally from the holistic motor planning evident in spontaneous speech, where entire phrases are temporally coordinated as integrated units, enabling Menzerath-like compression.

Studies of human speech production show that when instructed to produce sequences of real words or pseudowords, they exhibit advanced planning of whole sequences (Sternberg et al. 1990). These temporal patterns appeared similar when sequences consisted of different words or of the same word (Sternberg et al. 1978, 1988). This process is disrupted by our task demands where humans might be explicitly counting their vocalizations. Preventing nonverbal counting in humans through secondary interference tasks – such as rapid repetition of a word or finger tapping (Logie and Baddeley 1987; Trick 2005; Cordes et al. 2001) or by instructing rapid responses (Whalen et al. 1999) could reveal whether humans would exhibit temporal patterns more similar to crows when symbolic counting is suppressed. As we wanted the experimental paradigm for humans to be similar to that of the crows, we did not include such interference tasks. Future studies implementing these manipulations would be very interesting to test for divergence in strategy and temporal patterns in humans.

Additionally, as our crows have been trained over a longer period of time compared to the verbal instructions given to the humans just before recording, differences in these temporal patterns could also reflect practice in producing sequences containing a particular number of vocalizations. These recordings were collected shortly after crows learned the full task (Liao et al. 2024), and it would be interesting to examine data after additional experience; we speculate that with more familiarity, the balance between efficiency and skill might strengthen the relationship between sequence size and vocal durations.

### Call type composition and Menzerath’s law

In studies of Menzerath’s law across languages, the constituents are usually assumed to be qualitatively different. Though Menzerath’s law has been found in both the song motifs and repeat phrases of certain songbird species (James et al. 2021), one pattern across different studies is that homogenous vocalizations (repetitions of the same element) show less or no adherence to Menzerath’s law (Watson et al. 2020). In sequences with multiple call types, producing larger sequences could be achieved by increasing the number of shorter call types and/or reducing the duration of all call types (Gustison et al. 2016). Indeed, adherence to Menzerath’s law in the ecstatic display songs of African penguins was attributed to larger sequences having a far greater number of a short call type (Favaro et al. 2020).

In our numerically-cued vocal production task, though acoustic features varied with number cued and position in sequence, we assume the same type of vocalization was produced throughout the sequence for both crows and humans. It would be intriguing to perform clustering analyses on the different vocalizations in the wild crow vocal sequences, though this remains challenging due to variability in recording conditions and noise. A recent study found that vocal sequences of captive carrion, hooded, and hybrid crows have also been found to conform to Menzerath’s law (Wascher and Youngblood 2025) with varying strengths modulated by individual characteristics such as sex and age. Future comparisons of call acoustics across contexts could reveal whether the stronger Menzerath effect in wild versus trained crows is modulated by call type diversity. These constraints may also related to the different proposed functions of homogenous and heterogenous vocal sequences (Watson et al. 2020, 2024; Semple and McComb 2000), which are shaped by social and ecological constraints.

Our comparative approach highlights the importance of testing linguistic laws in controlled experimental settings that manipulate production demands. While examining communicative processes in the wild offers rich ecological validity, controlled tasks allow us to disentangle production constraints from communicative goals and probe the underlying mechanisms shaping vocal behavior. One limitation of our study is the smaller sample size of laboratory-trained crows and the lack of individual variability in age and sex compared to the wild population. We are also lacking contextual information from the wild crow recordings that could inform the emergence of temporal patterns involving different call types and sequence lengths. Overall, the differences between the vocal sequences of trained crows, wild crows, and humans demonstrate that expression of temporal patterns consistent with Menzerath’s law may emerge from an interaction between cognitive capacities, motor constraints, and context-specific demands.

## Supplementary Information

Below is the link to the electronic supplementary material.

Supplementary file1

## Data Availability

Data to reproduce analyses and figures are available on https://figshare.com/s/605f08b0e77a91d5fe58 with the DOI 10.6084/m9.figshare.31370020
